# High-contrast grating resonators for label-free detection of disease biomarkers

**DOI:** 10.1038/srep27482

**Published:** 2016-06-06

**Authors:** Tianbo Sun, Shu Kan, Gerard Marriott, Connie Chang-Hasnain

**Affiliations:** 1 Department of Electrical Engineering and Computer Science, University of California, Berkeley, CA 94720, USA; 2 Department of Bioengineering, University of California, Berkeley, CA 94720, USA.

## Abstract

A label-free optical biosensor is described that employs a silicon-based high-contrast grating (HCG) resonator with a spectral linewidth of ~500 pm that is sensitive to ligand-induced changes in surface properties. The device is used to generate thermodynamic and kinetic data on surface-attached antibodies with their respective antigens. The device can detect serum cardiac troponin I, a biomarker of cardiac disease to 100 pg/ml within 4 mins, which is faster, and as sensitive as current enzyme-linked immuno-assays for cTnI.

Point of care devices (POC) offer advantages over conventional technologies for biomarker detection including cost and speed, while requiring smaller amounts of sample and reagents. Since diagnostic tests account for an appreciable percentage of the national healthcare budget, the demand for more cost-effective POC devices and decentralized strategies for disease diagnostics will inevitably increase^1^,^2^,^3^,^4^,^5^,^6^,^7^,^8^,^9^,^10^,^11^,^12^,^13^,^14^.

In most cases, the amount of a target biomarker associated with a specific cancer, or neurologic or cardiovascular disease, is recorded within a POC device using an enzyme-linked immuno-assay (ELISA). The optical read-out may be as simple as a change in the absorption of light or the intensity of fluorescence of a dye that is produced by the antibody-linked enzyme^2^,^3^. Enzyme-antibody conjugates are produced by random chemical crosslinking of antibody and enzyme molecules, which produces a heterogeneous population of conjugates, some of which will exhibit a reduced affinity for the target biomarker. Moreover, a typical ELISA assay involves time-consuming incubation and wash-steps requiring fluidic control that increases the complexity and cost of the device. While ELISA-based detection dominates the field of POC devices, recent studies have shown label-free detection modalities offer an attractive alternative^7^,^8^,^9^,^10^,^11^,^12^,^13^. In particular, label-free optical sensing techniques have been described for POC devices including those that detect specific biomarkers using surface plasmon resonance^7^,^8^, photonic crystals^9^,^10^, integrated micro-cavities^11^,^12^, and metal nanohole arrays^13^. Although these devices allow for sensitive detection of target biomarkers, they are produced by expensive e-beam lithography and have limited utility for remote testing owing to the need for exact optical alignments that are usually carried out by specialized technicians.

Here we describe a new type of POC device that integrates a high-contrast grating (HCG) resonator for label-free detection of target biomarkers (Fig. 1(a,b))^15^,^16^. The high-contrast grating structure is shown to generate with very high efficiency, specific and strong resonances that are sensitive to changes in surface properties and can be monitored using surface-normal excitation^15^. The resonance quality factor (defined as resonance wavelength divided by the full width half maxima) of these devices is reported at ~3000 and has a refractive index sensitivity ~418 nm/RIU, both of which are higher than those reported for other nanostructure devices that also use direct surface-normal coupling^10^,^11^,^12^,^13^, including the guided-mode resonant filter^10^ and metal nanohole arrays^13^.

The binding of a biomolecule to the HCG surface results in a red-shift of the resonance wavelength, which is a consequence of the effective increase in the length of the optical cavity (Fig. 1(c)). A tunable laser is used as the excitation source and the reflected signal is recorded using a photodiode over a wavelength range of interest. The data is subsequently transferred to a computer for further processing (see Method). The HCG resonator has a narrow linewidth (0.5 nm) and exhibits a high sensitivity to changes in surface-binding, with even small changes in surface properties generating noticeable shifts in the resonance wavelength. The surface normal input of the probe light beam simplifies the optical alignment of the device, and allows us to use a low cost tunable laser and photodiode to measure molecular events at the surface. HCG’s are produced *en masse*, and at low cost using a standard silicon optical lithography and etching technique (see Method) that can accommodate 96-well, 384-well or 1536-well formats that may be suitable for high-throughput screening applications.

The HCG device exhibits a remarkable sensitivity to surface-binding events. This feature is exploited for the detection of biomarkers some of which are associated with human disease. This is achieved in part by chemically-linking detection antibodies against specific biomarkers to the HCG surface where they provide a platform for rapid, sensitive and specific detection of disease-associated antigens^17^. We are particularly interested in using the HCG device for label-free analysis of troponin I (cTnI), which is released into the blood from damaged cardiac muscle cells after myocardial infarction. cTnI in serum is a reliable and validated clinical marker of cardiac muscle tissue injury and stroke, which affects ~1 million people in the USA each year^18^,^19^,^20^,^21^. cTnI is often detected from blood samples using ELISA based platforms with read-out times of that can be as long as 10-hours. In this study we show how a HCG platform is used to generate quantitative analysis of cTnI over a clinically relevant concentration range of 100 pg/ml to 80 *μ*g/ml^17^ within 4 minutes.

## Results

### Design, fabrication and characterization of HCG resonators

**Figure 1:**
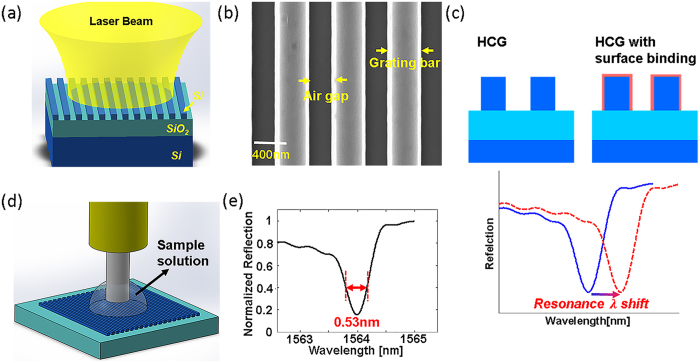
HCG resonator as a label-free biosensing platform. (**a**) Schematic of a surface normal coupled HCG resonator. (**b**) SEM image of gratings within a single fabricated HCG resonator. (**c**) Schematic of the principle of using HCG resonator as a protein binding sensing platform. The red-dashed curve indicates a red-shift in the resonance wavelength occurs when a protein binds to HCG surface. (**d**) A single mode fiber probe is immersed in the fluid above the HCG surface to avoid surface reflections. (**e**) A reflection spectrum recorded for a device having the configuration schematized in (**d**). A tunable laser centered at 1550 nm is used as excitation source and is run in a continuous sweeping mode. The reflection power is recorded in real time and is synchronized to laser sweeping rate.

The HCG is formed from an ultra-thin layer (a few hundred nm) of a silicon grating surrounded by a material of low refractive index. A schematic of a Si-based HCG resonator with surface-normal optical coupling is shown in Fig. 1(a). Unlike other gratings, the refractive index contrast in the HCG is high at both the entrance and exit planes. The large difference in refractive index (contrast) between the grating plane and its surroundings results in strong coupling between resonance modes at both the exit and entrance boundaries. Resonance is established inside the grating layer with the air/Si and Si/oxide interfaces acting as cavity boundaries. By choosing an appropriate grating period and grating bandwidth, the HCG is able to support only two resonance modes^16^. Moreover, by using a grating layer of appropriate thickness, the two resonance modes can be made to interfere constructively at the entrance and exit boundaries. This feature results in a high-Q resonator with the light propagating in a direction that is normal to the grating plane. This phenomenon has been described in earlier publications^15^,^16^; a brief summary of this principle is presented herein. We define *ρ* as the coupling coefficient matrix between the resonance modes at the entrance/exit boundaries, *φ* as the propagation factor inside the Si grating layer and *C* as the vector representing a self-sustainable mode that satisfies the round-trip condition. We can show that:


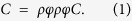


The intrinsic quality factor of HCG resonance is calculated by


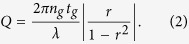


where *t*_*g*_ is the grating thickness, *λ* is the wavelength of the incident light, n_g_ is the group index, and *r* represents the eigenvalues of matrix *ρφ*^16^.

A detailed analysis of the design algorithm for the HCG resonator is described in Sections 1 & 4 of ref. 16. For HCG devices used in this study, the grating period is 792 nm, the bandwidth is 427 nm, and the grating thickness is 500 nm. An SEM image of gratings within a single device is shown in Fig. 1(b), with the measured dimension agreeing well with design value, as well as verifying the accuracy of our fabrication process.

A single mode fiber is used to excite resonances inside the HCG resonator. In practice, the fiber is adjusted vertically above the surface of the HCG to excite resonance – we show that this simple optical alignment results in accurate and robust measurement of reflection spectrum (Fig. 1(d). The fiber tip is immerged inside a target solution to avoid reflection at the surface. The reflection spectrum under fiber illumination of a HCG device immersed in phosphate buffered saline (PBS) is recorded as described in the Methods section, with a typical recording shown in Fig. 1(e). The quality factor determined from the reflection spectrum data shown in Fig. 1(e) is ~3000 with a center wavelength of 1564 nm.

**Figure 2:**
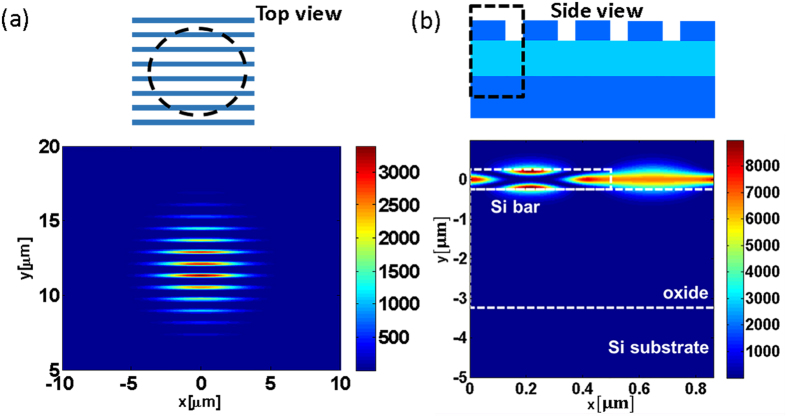
HCG resonator intensity pattern at resonance wavelength. (**a**) Top view of the resonance inside the HCG cavity excited by Gaussian beam with a 10 *μm* spot size at resonance wavelength (1564 nm). A large mode overlap with the input beam contributes to a simple and efficient coupling of the input light. (**b**) Side view of the resonance (showing one period) inside the HCG cavity. The resonance is designed to have large mode exposure to surroundings, which contributes to the high sensitivity of the device.

The HCG resonator has several advantages over other on-chip resonance-based devices for the detection of molecular events including an intrinsic ability to establish unique patterns of resonance modes. A simulated mode pattern at resonance for an HCG resonator excited with a Gaussian beam of ~10 *μm* spot size (output from a single mode fiber) is shown in Fig. 2(a). The top view of the resonance (at 1564 nm) sliced through the center of resonator is shown in Fig. 2(a). Efficient optical coupling of the input light to the HCG device is facilitated by the large mode overlap between the input Gaussian beam and the resonance mode. The actual sensing area is determined by the size of resonance pattern, and in our studies (illumination using a single mode fiber) it is approximately 40 *μ*m^2^. The side view of the resonance inside one grating period of the HCG is shown in Fig. 2(b), which reveals a key design feature in that the mode has a strong intensity at HCG surface. This feature results in strong interactions between the resonant light and surface-attached biomolecules.

### Sensitivity of the HCG device to changes in refractive index

**Figure 3:**
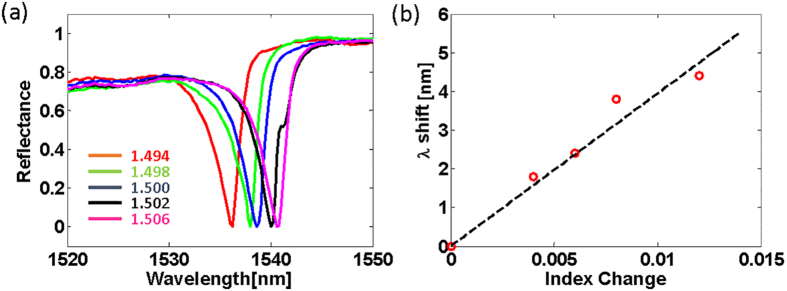
Quantification of HCG sensor sensitivity at different refractive indices. (**a**) Reflection spectra for a HCG resonator immersed in liquids with different refractive indices ranging from 1.494 to 1.506. (**b**) Resonance wavelength as function of the change in refractive index.

Next we carried out quantitative measurements on the intrinsic sensitivity of the HCG device to changes in refractive index. First we immersed the sensor surface with a liquid of known refractive index (RI), using solvents with a RI range from 1.494 to 1.506. The results of these measurements, shown in Fig. 3(a), show that an increase in RI results in a red-shift of the resonance wavelength. The shift in wavelength as a function of the change in RI, plotted in Fig. 3(b), reveals a linear relationship between ΔRI and the shift in the resonance wavelength. HCG sensors reported in this study have a RI sensitivity (RIS) of 418 nm/RIU (Refractive Index Unit), which is higher than corresponding sensitivities reported for other dielectric structures^9^,^10^. The high sensitivity of the HCG is attributed to the special resonance mode pattern of the HCG resonator.

### Quantitative analysis of antibody-antigen interactions using HCG sensors

**Figure 4:**
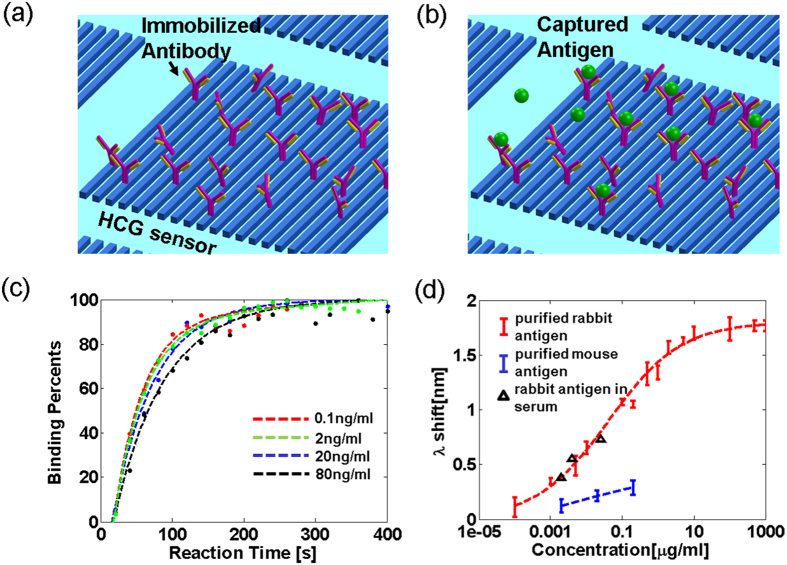
Three-dimensional schematic (not drawn to scale) and the experimental measurements illustrate the detection scheme using HCG resonator as platform for antibody-antigen binding assay. (**a**) HCG sensor immobilized with capturing antibody. (**b**) Antigen attaches to the antibody immobilized sensor and changes the surface properties of HCG sensor. (**c**) Real time recording of the interaction between surface bound goat anti-rabbit IgG and different concentrations of antigen (rabbit IgG). The dashed lines are the corresponding fits to data. (**d**) Red dots show the resonance wavelength shift for different concentrations of purified rabbit IgG added to a HCG resonator surface-coated with anti-rabbit IgG. The red dashed line is the fit to recorded data. Blue dots show that the incubation of mouse IgG with surface linked anti-rabbit IgG results in smaller shift of the resonance wavelength. The black triangles show the response of the HCG sensor to the addition of rabbit IgG dissolved in goat serum (Sigma). The bars indicate the error range for the 3 measurements.

To characterize the sensitivity of the HCG sensor in detecting target antigens, we tested a complementary antibody-antigen pair composed of unlabeled IgG from rabbit serum (Sigma, I5006) as the antigen, and a polyclonal antibody directed against rabbit IgG that was produced in goat (Sigma; R2004) as the detection antibody. A schematic of the antibody-antigen binding platform is shown in Fig. 4(a,b). First the surface of the HCG sensor is coupled covalently with the anti-rabbit IgG antibody via a maleimide-thiol coupling reaction (see Methods). The spectral response of the sensor is recorded before adding rabbit IgG, with the resonance wavelength for the measurement assigned as the reference. Next solutions (200 *μ*l) of the rabbit IgG (antigen) are added separately and sequentially to the HCG surface over a concentration range from 0.1 ng/ml to 1 mg/ml. The reflection spectrum is recorded after the addition of each antigen sample.

Spectral data showing an interaction between surface-bound goat anti-rabbit IgG and rabbit IgG studies are presented in Fig. 4(c,d). A standard curve for the device is generated by recording the spectral shift for different concentrations of the purified antigen in PBS; three parallel titrations are performed for each antigen concentration to improve the precision of the measurement. The standard curve, recorded over a 1000-fold range of antigen concentration, shows the red-shift in the resonance wavelength is proportional to the concentration of antigen. The signal associated with antigen binding to the surface-coupled antibody saturates at ~3 *μ*g/ml. We established the lowest detectable concentration of antigen recorded for this device at 100 pg/ml, which resulted in a red-shift of the resonance wavelength of 60 pm (red trace of Fig. 4(d)). The specificity of the interaction between complementary antibody-antigens is demonstrated using a negative control composed of a non-complementary antibody-antigen pair, with data for surface bound anti-rabbit IgG and mouse IgG shown in the blue line of Fig. 4(d). The shift in the wavelength for this control sample is at least three times smaller compared to the complementary rabbit IgG/anti-rabbit IgG system. The device worked equally well for the complementary rabbit IgG/anti-rabbit IgG pair where the rabbit IgG was dissolved in serum rather than PBS, which suggests that a low level of non-specific binding of serum proteins to the HCG surface. Three known concentrations of rabbit IgG dissolved in serum (2 ng/ml, 10 ng/ml and 80 ng/ml) lie close to their expected values on the standard curve (black triangles in Fig. 4(d)).

To demonstrate the ability of the HCG device to record dynamic interactions between rabbit IgG and anti-rabbit IgG, we recorded the time-dependence of the shift in the resonance wavelength after adding rabbit IgG to the anti-rabbit IgG labeled surface. Figure 4(c) shows the normalized surface binding curves as function of time, which were obtained by integrating the difference of the spectral response (shift of resonance wavelength) for the started time and that recorded at a given time. In this study the surface binding response of the HCG device was recorded continuously after the addition of 4 different concentrations of the antigen. As shown in Fig. 4(c), the signal associated with antigen binding increases exponentially with a time constant of ~50 sec (on average), and reaches a plateau at 95% of the equilibrium value within 200 seconds. In conclusion, we have shown the HCG device allows for rapid (within 4 minutes) and quantitative analyses of the amount of a target antigen in buffer and serum, and shown the device can also calculate kinetic constants associated with complex formation (or dissociation).

### Quantitative analysis of cardiac Troponin I

**Figure 5:**
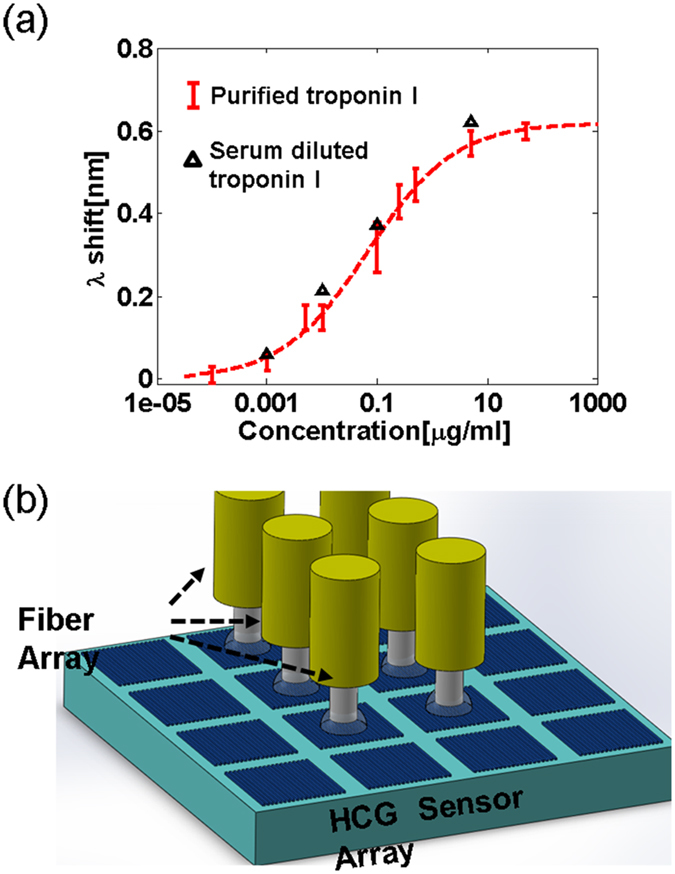
(**a**) Measurement results for troponin I molecule inside PBS (red dots) and serum (black triangles). The bars indicate the error range over 3 measurements. (**b**) HCG sensor array combined with fiber array for high throughput screening.

Purified human cTnI^19^ is detected in serum and PBS using a commercial anti-human troponin I antibody that binds to an epitope between residues 86–209 on troponin I (sc-133117, Santa Cruz Biotechnology). The monoclonal antibody is covalently linked to the HCG surface using the maleimide-thiol coupling strategy described in the Methods section. The standard curve for cTnI binding to anti-cTnI on the HCG surface is generated by measuring the change in the resonance wavelength for different dilutions of purified cTnI in PBS (0.1 ng/ml to 80 *μ*g/ml; Fig. 5(a)). Purified cTnI is also measured on the HCG device after being diluted at 4 defined concentrations in fetal bovine serum. The concentrations of cTnI in these serum samples are determined by comparing their spectral shifts to a standard curve (black triangles in Fig. 5(a)). Our studies show the HCG device is useful for rapid (4 minutes) and quantitative determinations of cTnI in PBS and in mock serum samples over a concentration range that is relevant for clinical analysis of cTnI in the serum of stroke victims (100 pg/ml to 80 *μ*g/ml)^17^.

## Discussion

Devices and detection systems that allow for quantitative, sensitive and accurate determinations of validated biomarkers at levels associated with early detection of a disease are highly valued in clinical diagnoses. The most popular approach to quantify specific biomarker in POC devices is based on the ELISA. ELISA platforms however are slow, and require complex fluidic control for multi-step processing of the sample and detection reagents, the time to complete an ELISA assay is usually on the order of 4–12 hours^22^. Moreover, ELISAs employ at least two coupled antibodies, the capture antibody mobilized on the microwell surface and a detection antibody that is conjugated with a florescent dye or an enzyme that chemically transforms a substrate into a colored or chemiluminescent product for readout, the performance of which vary for particular manufacturers and in different batches^22^,^23^. In this study we described a high-contrast grating (HCG) resonator that allows for label-free detection and quantification of interactions between only one surface bound antibody and its target antigen, shorten the running time to 4 minutes, at the same time achieving comparable detection limits (100 pg/ml) to reported commercial ELISA kits and the theoretical detection limit of a HRP/TMB-based ELISA assay^24^. We also provide a performance comparison between the HCG sensor and other reported label free sensor technologies in Table 1. The HCG device compares favorably with other technologies that have been applied to the detection of biomarkers such as cTnI in terms of detection sensitivity (100 pg/ml to 80 *μ*g/ml)^17^ and sensing area. Moreover, we have shown the HCG device is relatively insensitive to the non-specific binding of serum proteins, which is a critical requirement in the development of clinically relevant assays of disease biomarkers. Moreover the HCG device offers additional benefits that include speed, with assays typically complete within 4 minutes), feasibility of mass production, Si CMOS compatibility, and suitability for remote detection *eg* in the home or in a doctor’s office. The low-cost HCG device is simple to construct and to operate, and allows for sensitive and robust, surface-based detection formats to quantify disease biomarkers at disease-relevant concentrations within 4 minutes. We further showed that cTnI can be detected in mock serum samples with a sensitivity of 100 pg/ml, which is sensitive enough for the detection of serum cTnI levels associated with acute myocardial infarction (AMI), and compares well with the sensitivity obtained using POC devices (>400 pg/ml)^17^,^25^. The simple design of the HCG sensor, which is based on a standard silicon manufacturing process, allows for the fabrication of more than 10000 unique sensors on a single cm^2^ chip that can be further embellished with integrated microfluidic and electronic systems. The integration of a stand-alone tunable laser and photodiode helps to reduce the cost of the measurement system, while the surface-normal fiber detection format overcomes a requirement in other competing devices for continuous adjustments to maintain a precise optical alignment. A simple extension of the HCG platform described in this study would be to combine a HCG sensor array with a fiber array, as shown in Fig. 5(b). This new platform could facilitate high-throughput, parallel screening of biomarkers in thousands of samples.

## Methods

### Fabrication method

The devices are patterned onto a 6 inch silicon-on-insulator with Si-layer thickness 500 nm wafer (from SOITEC) using DUV (ASML 300) lithography followed by silicon refractive ion etching (Lam Research), which are both standard processes in semiconductor manufacturing.

### Reflection spectrum measurements

A tunable laser (HP 8164) centered at 1550 nm is used as the excitation and is set in a continuous sweeping mode between 1560 nm and 1565 nm at a rate of 40 nm/sec. The power of the reflected light is recorded using a photodiode in real time and is synchronized to the laser sweep rate through an Oscilloscope. Data is then transferred into a computer for further process.

### Antibody immobilization

Maleimide groups are introduced to the antibody by treating the antibody (1 mg/ml) with 100 *μ*M maleimidobenzoic acid succinimide ester (MBS, Sigma) in PBS for 2 hours at room temperature. The mixture is then passed over a PD-10 column in PBS to remove unlabeled MBS and fractions containing the MBS-antibody conjugate are pooled. The surface of the Si-sensor is thiol-silanized using a fresh 5% solution of (3-Mercaptopropyl) trimethoxysilane (MPTMS) in IPA for 1 hr. After washing with PBS the surface is treated with a 1mg/ml of the MBS-antibody. The reaction between the MBS-antibody conjugate and surface thiol groups on the HCG results in a stable thioether bond. After an incubation period of two hours, the surface is washed with PBS, and a blocking buffer containing 3%BSA is applied to the surface to reduce the non-specific binding of serum proteins.

## Additional Information

**How to cite this article**: Sun, T. *et al*. High-contrast grating resonators for label-free detection of disease biomarkers. *Sci. Rep*. **6**, 27482; doi: 10.1038/srep27482 (2016).

## Figures and Tables

**Table 1:** Comparison of label-free biosensor in biomarker detection^10^.

Device	Sample	Sensitivity [pg/ml]	Sensing Area [μm^2^]	Manufacturing	Test Specification
SPR^8^	IgG	40	~10^5^	Not Si compatible	Prism coupler
Au nanoparticle^7^	BSA	4	~1	Not Si compatible; Nonuniformity	Low detection efficiency
CNT^5^	IgG	1	~6	Low yield	Fragile
Nanolaser^10^	BSA	17	<1	Not Si compatible; E-beam required	High NA optics
Si ring resonator^11^	BSA	10^4^	~60	Si CMOS compatible; Optical Litho.	Angled Fiber I/O + grating coupler
HCG resonator	IgG	100	~40	Si CMOS compatible; Optical Litho.	Surface normal Fiber I/O with high efficiency
